# Predicting Response to Exclusive Combined Radio-Chemotherapy in Naso-Oropharyngeal Cancer: The Role of Texture Analysis

**DOI:** 10.3390/diagnostics14101036

**Published:** 2024-05-17

**Authors:** Eleonora Bicci, Leonardo Calamandrei, Antonio Di Finizio, Michele Pietragalla, Sebastiano Paolucci, Simone Busoni, Francesco Mungai, Cosimo Nardi, Luigi Bonasera, Vittorio Miele

**Affiliations:** 1 Department of Radiology, Azienda Ospedaliero-Universitaria Careggi, 50134 Florence, Italy; fmungai@gmail.com (F.M.); luigi.bonasera72@gmail.com (L.B.); vmiele@sirm.org (V.M.); 2 Department of Experimental and Clinical Biomedical Sciences, Radiodiagnostic Unit n. 2, University of Florence, Azienda Ospedaliero-Universitaria Careggi, Largo Brambilla 3, 50134 Florence, Italy; leonardo.calamandrei@unifi.it (L.C.); antonio.difinizio@unifi.it (A.D.F.) cosimo.nardi@unifi.it (C.N.); 3 Department of Radiology, Ospedale San Jacopo, Via Ciliegiole 97, 51100 Pistoia, Italy; michele.pietragalla@unifi.it; 4 Department of Health Physics, L.Go Brambilla, Careggi University Hospital, 50134 Florence, Italy; sebastiano.paolucci@studenti.unicam.it (S.P.); busonis@aou-careggi.toscana.it (S.B.)

**Keywords:** texture analysis, head and neck, naso-oropharyngeal tumors, HPV, EBV, magnetic resonance imaging

## Abstract

The aim of this work is to identify MRI texture features able to predict the response to radio-chemotherapy (RT-CHT) in patients with naso-oropharyngeal carcinoma (NPC-OPC) before treatment in order to help clinical decision making. Textural features were derived from ADC maps and post-gadolinium T1-images on a single MRI machine for 37 patients with NPC-OPC. Patients were divided into two groups (responders/non-responders) according to results from MRI scans and 18F-FDG-PET/CT performed at follow-up 3–4 and 12 months after therapy and biopsy. Pre-RT-CHT lesions were segmented, and radiomic features were extracted. A non-parametric Mann–Whitney test was performed. A *p*-value < 0.05 was considered significant. Receiver operating characteristic curves and area-under-the-curve values were generated; a 95% confidence interval (CI) was reported. A radiomic model was constructed using the LASSO algorithm. After feature selection on MRI T1 post-contrast sequences, six features were statistically significant: gldm_DependenceEntropy and DependenceNonUniformity, glrlm_RunEntropy and RunLengthNonUniformity, and glszm_SizeZoneNonUniformity and ZoneEntropy, with significant cut-off values between responder and non-responder group. With the LASSO algorithm, the radiomic model showed an AUC of 0.89 and 95% CI: 0.78–0.99. In ADC, five features were selected with an AUC of 0.84 and 95% CI: 0.68–1. Texture analysis on post-gadolinium T1-images and ADC maps could potentially predict response to therapy in patients with NPC-OPC who will undergo exclusive treatment with RT-CHT, being, therefore, a useful tool in therapeutical–clinical decision making.

## 1. Introduction

Head and neck cancers are the seventh most common malignancies worldwide [1,2]. Among these, carcinoma of the oropharynx (OPC) represents the most common tumor in HPV-endemic areas [3], while nasopharyngeal carcinoma (NPC) is endemic in North Africa and South East Asia [4]. Tumor histotypes are classified into type 1 SCC keratinizing, type 2 (differentiated non-keratinizing), and type 3 (undifferentiated) [5]. Oropharynx squamous cell carcinoma (OPSCC) has historically been linked to smoking and alcohol consumption as the main risk factors, but in the last 20 years, the increase in incidence has been driven by the spread of infection by the human papilloma virus (HPV), becoming an important risk factor, given the oncogenic capabilities of some of the most widespread viral genotypes [6]. OPSCC HPV+ tumors present some peculiarities; they mainly affect patients of a high socio-economic status, males, and those aged 40–60 years old, and they are generally located in the tongue and palatine tonsil [7,8]. Also, they correlate less with smoking and alcohol and have a favorable prognosis compared to their HPV- counterparts [9]. Nasopharyngeal squamous cell carcinoma (NPSCC) has been classified by the WHO into three subtypes, as type 2 (differentiated non-keratinizing) and type 3 (undifferentiated), in which the role in the pathogenesis of the Epstein–Barr virus (EBV) is known, while for type 1, the pathogenesis is unclear and the role of HPV is suspected [4]. The tumor generally originates in the fossa of Rosenmüller, at the level of the posterolateral wall of the pharynx [10]. A diagnosis of OPS and NPC requires a histological examination, which allows for a characterization of the tumor, as well as the evaluation of viral DNA or proteins that correlate with infection status and prognosis, such as p16 and p53 [11,12]. Samples are taken through multiple biopsies after an endoscopic evaluation of the primary lesion or lymph nodes [13]. Guidelines indicate that tumor size measurement and staging, histological grading, molecular patterns, and lymph node involvement or metastasis are essential prerequisites for a valid prognostic evaluation of the patient [14,15,16]. There are different therapeutic strategies and different prognoses associated with each tumor type [17,18,19]. This was further highlighted in the eighth UICC/AJCC staging system, where two different TNM staging systems are proposed depending on the expression of p16 [20,21]. Magnetic resonance imaging (MRI), computed tomography (CT), and positron emission tomography (PET-CT) are used for the staging and follow-up of head and neck tumors; ultrasound is used when latero-cervical lymph node metastases need to be assessed [22,23,24]. Among these, MRI is the most suitable exam for better visualizing soft tissues, as well as performing a multiparametric evaluation of lesions, thanks to the use of dynamic contrast-enhanced perfusion imaging (DCE-PWI) and diffusion weighted imaging (DWI) [25,26,27]. Being able to non-invasively evaluate characteristics specifically associated with one or the other tumor subtype through imaging is of paramount importance. For example, HPV+ tumors are generally associated with exophytic growth, well-defined border enhancement, and cystic-appearing lymph node metastases, unlike HPV- tumors, in which the primary lesion often has poorly defined margins [28,29,30]. However, a purely morphological evaluation of those differences is not sufficient due to the overlap of some common characteristics between the HPV+ and HPV- lesions [31]. Texture analysis is an application of radiomics based on the mathematical analysis of the spatial distribution of pixel values within a region of interest (ROI) of a radiological image, which allows for obtaining quantitative information on tissue heterogeneity not otherwise perceivable by the human eye [32,33]. The existence of radiomic features related to different tumor histotypes, different prognoses, and different risks in terms of recurrence or response to therapy makes its application in OPSCC and NPSCC tumors an interesting and promising field of research [34,35,36,37,38,39,40].

## 2. Materials and Methods

### 2.1. Patient Selection

This is a single-center, observational, retrospective study. Patients with a histological diagnosis of HPV-related OPC or EBV-related NPC who underwent an MRI examination between January 2014 and January 2024 in AOUC Careggi were enrolled.

Inclusion criteria were as follows:

- Over 18 years of age;
- Diagnosis of HPV + OPC or EBV + NPC;
- Having undergone RT-CHT as an exclusive treatment;
- Pre-treatment MRI examination available.

Exclusion criteria were as follows:

- Previous exposure to radiation therapy in the head and neck district;
- Previous surgery in the head and neck district;
- MRI examination performed in a different center or on a different machine;
- No ADC maps or T1w post-contrast sequences;
- MRI not performed, neither for tumor staging nor after the 3–4-month follow-up;
- No cross-sectional imaging follow-up, including both FDG-PET/TC and MRI, or carried out for less than 12 months.

**Figure 1:**
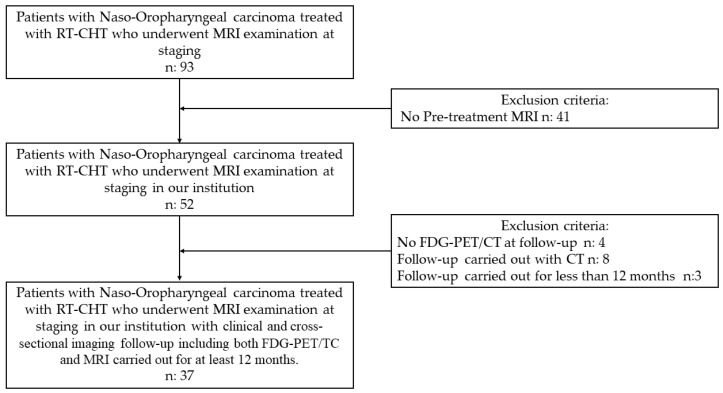
Workflow of patient selection following inclusion and exclusion criteria.

The patient selection algorithm is shown in Figure 1. Starting from a population of 93 patients, 41 were excluded because no pre-treatment MRI was performed in our hospital. Of the remaining 52, 4 patients were not studied with FDG-PET/CT and 8 patients underwent follow-up with CT instead of MRI. In total, 3 patients carried out follow-up for less than 12 months. The final sample included 37 patients (16 women and 21 men; mean age, 59 years; median age, 58.5 years; range, 36–81 years); 29 patients were affected by OPC and the remaining 8 patients were affected by NPC.

### 2.2. Image Acquisition

MRI examinations were performed with 1.5 T Magnetom Aera (Siemens Healthcare, Erlangen, Germany). Please refer to Table A1 in Appendix A for information about image acquisition parameters and the sequences employed.

### 2.3. Image Analysis

**Figure 2:**
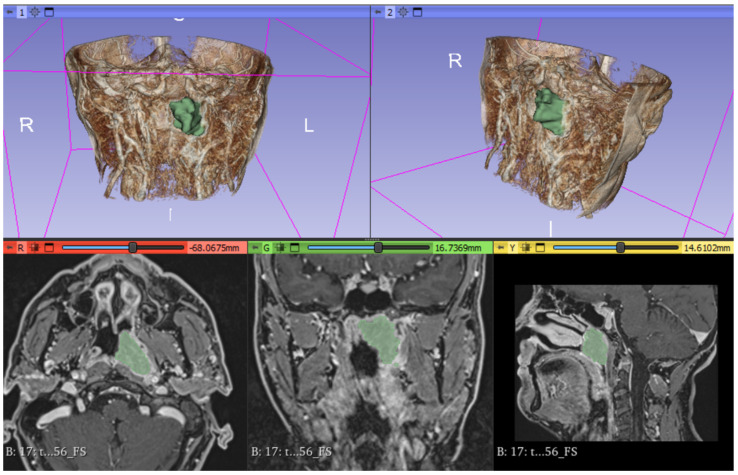
Segmentation of the lesion on 3D Slicer software. The volume of interest (VOI) is traced in green. The 3D render shows tumor mass in green.

From pre-treatment MRI scans, the entire volume of the lesion was segmented on both ADC maps and post-gadolinium T1w sequences by employment of the open-source software 3DSlicer (software version 4.10.2, https://www.slicer.org/ accessed on 18 December 2023). The ROI (region of interest) was delineated slice-by-slice for each patient by a radiologist with an experience of over 5 years in head and neck pathology (Figure 2).

Textural features were extracted by means of the SlicerRadiomics plug-in for 3D slicer. A total of 107 textural features, subdivided into First Order, 3D Shape-Based, Gray Level Co-occurrence Matrix (GLCM), Gray Level Size Zone Matrix (GLSZM), Gray Level Run Length Matrix (GLRLM), Neighboring Gray Tone Difference Matrix (NGTDM), and Gray Level Dependence Matrix (GLDM) classes were extracted according to PyRadiomics—an open-source python package for the extraction of radiomics data from medical images.

#### Division into Groups Based on Imaging and Histological Examination

Patients were divided into two groups based on MRI, FDG-PET/CT, and biopsy results. The “‘Positive” group (group 0) included patients with a persistence or recurrence of disease after RT-CHT; the “‘Negative” group (group 1) included responder patients, with no residual cancer after RT-CHT.

- Group 1: persistence or recurrence of disease—residual cancer—10 patients. Mass-like lesions showing intermediate signal intensity (SI) on T2w imaging; restricted diffusion with a subsequent decrease in ADC values, non-homogeneous post-contrast enhancement; positive FDG-PET/TC; positive biopsy;
- Group 2: responder patients—non residual cancer—27 patients. No mass at follow-up or mass-like lesion with negative biopsy (inflammatory oedema or fibrosis).

### 2.4. Statistical Analysis

The 107 features extracted from both ADC maps and post-contrast T1w images were first studied by employing a Shapiro–Wilk test to determine the nature of the distribution of data among the two groups of patients.

Features with a Gaussian distribution (*p*-value > 0.05 on Shapiro–Wilk test) were further studied by employing the parametric *t*-test, whereas features that showed a non-Gaussian distribution (*p*-value < 0.05 on Shapiro–Wilk test) were studied with the non-parametric Mann–Whitney test. The resulting *p*-values from these tests were used to determine if any distribution varied in a statistically significant way (*p*-value < 0.05) from one group to the other.

Subsequently, a radiomics model was produced using a LASSO (least absolute shrinkage and selection operator) logistic regression model. The LASSO model was applied to the totality of the 107 features, regardless of statistical significance. Receiver operating characteristic curves (ROCs) were charted; the penalty coefficient λ of the LASSO model was optimized in order to obtain the maximum area under the curve (AUC) by employing a 10-fold cross-validation technique via minimum criteria.

The same process was independently applied to ADC map data and to post-contrast T1w data.

## 3. Results

### 3.1. ADC Maps

On the ADC maps, 16 features diverged in a statistically significant way between the two groups of patients (see Table 1 for the full list), indicated as follows:

- 9 First order features;
- 4 Gray Level Co-occurrence Matrix features;
- 1 Gray Level Dependence Matrix feature;
- 2 Gray Level Size Zone Matrix features.

**Table 1:** Significative features on ADC maps.

Significative Features	Test	*p*-Value
firstorder_10Percentile	*t*-test	0.020
firstorder_90Percentile	*t*-test	0.022
firstorder_Energy	Mann–Whitney test	0.044
firstorder_InterquartileRange	Mann–Whitney test	0.048
firstorder_Maximum	*t*-test	0.040
firstorder_Mean	*t*-test	0.017
firstorder_Median	*t*-test	0.048
firstorder_RootMeanSquared	*t*-test	0.017
firstorder_TotalEnergy	Mann–Whitney test	0.037
glcm_DifferenceEntropy	*t*-test	0.044
glcm_JointEnergy	Mann–Whitney test	0.031
glcm_JointEntropy	*t*-test	0.042
glcm_SumEntropy	*t*-test	0.028
gldm_SmallDependenceLowGrayLevelEmphasis	*t*-test	0.017
glszm_GrayLevelNonUniformityNormalized	*t*-test	0.037
glszm_ZoneEntropy	*t*-test	0.040

Of these, the most significant ones were firstorder_10Percentile, firstorder_90Percentile, firstorder_Mean firstorder_RootMeanSquared, and gldm_SmallDependenceLowGrayLevelEmphasis.

Then, a LASSO logistic regression model was applied to the totality of the 107 features. The features that yielded the best performance in the distinction between the two groups of patients, according to LASSO, were the following:

- shape_Elongation;
- firstorder_10Percentile;
- glcm_ClusterShade;
- glcm_SumEntropy.

It is interesting to note that LASSO selected two features that did not vary significantly between the two groups, shape_Elongation and glcm_ClusterShade.

**Figure 3:**
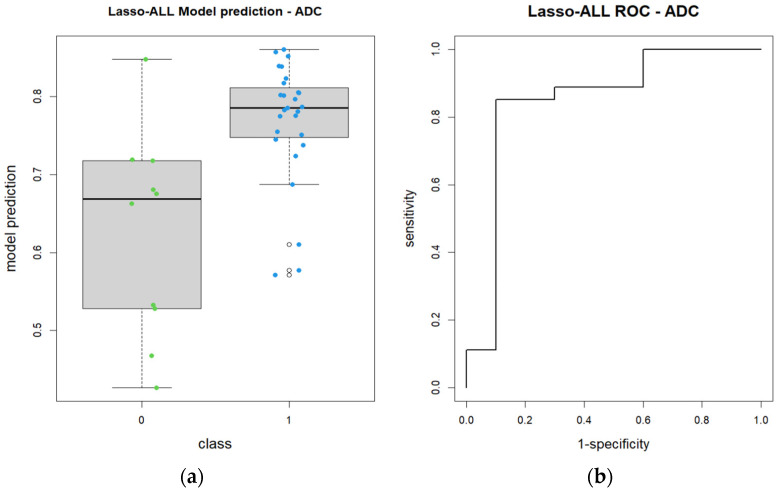
Lasso model for the ADC maps: (**a**) box plot graph showing performance of the model; (**b**) ROC curve for the Lasso model.

A ROC curve was then charted with 95% CI = 0.6709–1 (DeLong). The AUC was 0.8481 (Figure 3).

A box plot graph was then charted to show the performance of the model (Figure 3). The radiomics model was capable of accurately identifying patients as belonging to their respective group, as shown by the high AUC and box plot (Figure 3).

### 3.2. Post-Contrast T1w Imaging

On post-contrast T1w imaging, 43 features diverged in a statistically significant way between the two groups of patients (see Table 2 for the full list), indicated as follows:

- 8 First order features; 6 3D Shape-Based;
- 14 Gray Level Co-occurrence Matrix (GLCM);
- 5 Gray Level Size Zone Matrix (GLSZM);
- 5 Gray Level Run Length Matrix (GLRLM);
- 2 Neighboring Gray Tone Difference Matrix (NGTDM);
- 3 Gray Level Dependence Matrix (GLDM).

**Table 2:** Significative features on post-contrast T1w imaging.

Significative Features (43)	Test	*p*-Value
shape_LeastAxisLength	*t*-test	0.0053
shape_MeshVolume	Mann–Whitney test	0.0082
shape_MinorAxisLength	*t*-test	0.0437
shape_SurfaceArea	Mann–Whitney test	0.0127
shape_SurfaceVolumeRatio	Mann–Whitney test	0.0114
shape_VoxelVolume	Mann–Whitney test	0.0082
firstorder_Energy	Mann–Whitney test	0.0031
firstorder_Entropy	*t*-test	0.0068
firstorder_InterquartileRange	*t*-test	0.0210
firstorder_MeanAbsoluteDeviation	*t*-test	0.0173
firstorder_RobustMeanAbsoluteDeviation	*t*-test	0.0155
firstorder_TotalEnergy	Mann–Whitney test	0.0102
firstorder_Uniformity	Mann–Whitney test	0.0058
firstorder_Variance	Mann–Whitney test	0.0141
glcm_ClusterProminence	Mann–Whitney test	0.0282
glcm_ClusterTendency	Mann–Whitney test	0.0092
glcm_Contrast	*t*-test	0.0362
glcm_Correlation	*t*-test	0.0104
glcm_DifferenceAverage	*t*-test	0.0360
glcm_DifferenceEntropy	Mann–Whitney test	0.0212
glcm_DifferenceVariance	*t*-test	0.0352
glcm_Imc2	*t*-test	0.0161
glcm_InverseVariance	*t*-test	0.0456
glcm_JointEnergy	Mann–Whitney test	0.0127
glcm_JointEntropy	*t*-test	0.0071
glcm_MaximumProbability	Mann–Whitney test	0.0309
glcm_SumEntropy	*t*-test	0.0046
glcm_SumSquares	Mann–Whitney test	0.0102
gldm_DependenceEntropy	*t*-test	0.00028
gldm_DependenceNonUniformity	Mann–Whitney test	0.0021
gldm_GrayLevelVariance	Mann–Whitney test	0.0141
glrlm_GrayLevelNonUniformity	Mann–Whitney test	0.0405
glrlm_GrayLevelNonUniformityNormalized	Mann–Whitney test	0.0051
glrlm_GrayLevelVariance	Mann–Whitney test	0.0127
glrlm_RunEntropy	*t*-test	0.0021
glrlm_RunLengthNonUniformity	Mann–Whitney test	0.0024
glszm_GrayLevelNonUniformity	Mann–Whitney test	0.0058
glszm_GrayLevelNonUniformityNormalized	Mann–Whitney test	0.0082
glszm_GrayLevelVariance	Mann–Whitney test	0.0212
glszm_SizeZoneNonUniformity	Mann–Whitney test	0.0024
glszm_ZoneEntropy	*t*-test	0.00074
ngtdm_Coarseness	Mann–Whitney test	0.0114
ngtdm_Strength	*t*-test	0.0208

Among these, the most significant ones were gldm_DependenceEntropy, gldm_DependenceNonUniformity, glrlm_RunEntropy, glrlm_RunLengthNonUniformity, glszm_SizeZoneNonUniformity, and glszm_ZoneEntropy.

Again, a LASSO logistic regression model was applied to the totality of the 107 features. The features that yielded the best performance in the distinction between the two groups of patients, according to LASSO, were the following:

- gldm_DependenceNonUniformity;
- gldm LargeDependenceLowGrayLevelEmphasis;
- glrlm_RunEntropy;
- ngtdm_Strength.

Once again, it is interesting to note that feature gldm_LargeDependenceLowGrayLevelEmphasis was not statistically significant according to *p*-value.

**Figure 4:**
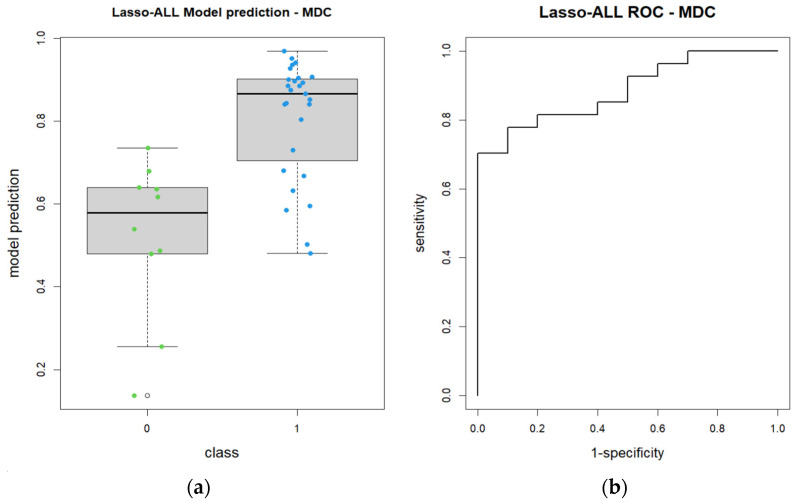
Lasso model for post-contrast T1w imaging: (**a**) box plot graph showing performance of the model; (**b**) ROC curve for the Lasso model.

A ROC curve was then charted with 95% CI = 0.7802–0.9902 (DeLong). The AUC was 0.8852 (Figure 4).

A box plot graph was then charted to show the performance of the model (Figure 4). The radiomics model showed a much better performance in the identification of patients belonging to group 1 as opposed to the same model applied to ADC maps, as shown by the box plot graph.

Both datasets were also combined to develop a third LASSO logistic regression model, which showed overall worse performance than both the ADC map model and the post-contrast T1w model and was, therefore, not studied any further.

## 4. Discussion

This current study represents a challenge—to use texture analysis of MRI images to predict the response to exclusive combined RT-CHT in patients with naso-oropharyngeal carcinoma before treatment.

To the best of our knowledge, only a few studies in the literature have focused on the assessment of local treatment outcomes for OPC and NPC based on locally derived radiomics-based image analysis in contrast-enhanced MRI images and ADC maps. Furthermore, in this study, a single MRI machine was used to study the totality of the patients, to reduce feature heterogeneity.

In the era of personalized medicine, innovative approaches based on imaging features able to quantitatively classify tumor phenotypes and aggressiveness by the employment of non-invasive techniques is crucial. To this end, the addition of quantitative imaging analysis, such as intra-lesional heterogeneity, to routinely acquired imaging could represent a great opportunity to obtain fundamental information to further stratify patient risk and expected outcomes.

In this context, this study sought to evaluate the correlation between the local textural features of primary tumors of the naso-oropharynx and local control of disease and outcome.

Among the studies that have evaluated the response to therapy of head and neck tumors, the work of Cozzi et al. [41] analyzed how textural features relate to the local control of disease after RT-CHT using computed tomography imaging, and it showed promising results.

A study by Haider et al. [42] tried to apply machine learning algorithms to combined PET and non-contrast CT by extracting radiomic features from baseline clinical scans for the prediction and risk stratification of post-radiotherapy in HPV-associated, OPC-affected patients.

In this study, a similar approach was employed on MRI sequences. Post-contrast imaging was selected, as more aggressive tumors tend to show enhancement patterns marked by a greater heterogeneity secondary to the presence of larger areas of intralesional necrosis, also often due to increased tumor size. On the other hand, low-grade tumors often show more intense and homogeneous enhancement.

ADC maps were also studied to infer further information regarding intra-tumoral changes in cellularity and the presence of areas of necrosis.

### 4.1. Post-Contrast T1w Imaging

In this analysis, six textural features on post-gadolinium T1 images (DependenceEntropy, DependenceNonUniformity, RunEntropy, RunLengthNonUniformity, SizeZoneNonUniformity, ZoneEntropy) differed in a statistically significant manner between the two groups of patients (tumor persistence/recurrence post RT-CHT and full response, respectively).

DependenceEntropy and DependenceNonUniformity belong to the Gray Level Dependence Matrix (GLDM) subgroup. GLDM quantifies gray level dependencies in an image, and particularly the measure of similarity of dependency throughout the image, with a lower value indicating more homogeneity among dependencies in the image. In our study, these features showed higher values among patients with a persistence/recurrence of disease after therapy, therefore demonstrating a greater structural heterogeneity in lesions unresponsive to therapy.

RunEntropy and RunLengthNonUniformity belong to the Gray Level Run Length Matrix (GLRLM). GLRLM quantifies gray level runs, which are defined as the length in number of consecutive pixels that have the same gray level value. RunLengthNonUniformity measures the similarity of run lengths throughout the image, with a lower value indicating more homogeneity among run lengths in the image, while RunEntropy measures the uncertainty/randomness in the distribution of run lengths and gray levels. A higher value indicates more heterogeneity in the texture patterns. Likewise, higher values of these features, seen in more heterogeneous lesions, are associated with a lower response to treatment.

SizeZoneNonUniformity and ZoneEntropy belong to the Gray Level Size Zone (GLSZ). GLSZ quantifies gray level zones in an image. A gray level zone is defined as the number of connected voxels that share the same gray level intensity. SizeZoneNonUniformity measures the variability of size zone volumes in the image, with a lower value indicating more homogeneity in size zone volumes, while ZoneEntropy measures the uncertainty/randomness in the distribution of zone sizes and gray levels. A higher value indicates more heterogeneity in the texture patterns. These features showed different values in the two different groups, with the “Positive” group demonstrating higher values because of the greater textural heterogeneity of more aggressive and less responding histotypes.

**Figure 5:**
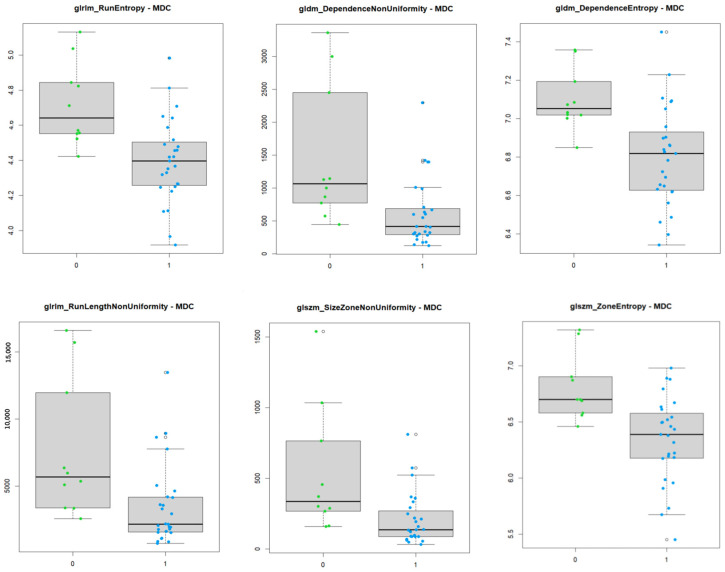
Box plot showing the different distribution of values of significant textural features among the two groups of patients on post-contrast T1w imaging.

These findings are better visualized in the box plot graphs showing the distribution of feature values among the two groups (Figure 5). Note the difference between group 0 and group 1 and the overall higher values of the former when compared to the latter.

These findings are in agreement with the actual greater textural heterogeneity of more aggressive tumors. Higher values correlate with a worse response to RT-CHT. Further confirmation is given by the LASSO radiomics model, showing an overall high AUC (Figure 4) and predictive performance when assigning patients to group 0, depending on significant feature values.

### 4.2. ADC Maps

Five textural features differed in a statistically significant manner on ADC maps, with these being firstorder_10Percentile, firstorder_90Percentile, firstorder_Mean, firstorder_RootMeanSquared, and gldm_SmallDependenceLowGrayLevelEmphasis, most of which—with the exception of gldm_SmallDependenceLowGrayLevelEmphasis—are part of the first-order group.

Furthermore, all of the significant first-order features were part of the histogram feature subgroup. While the first order overall describes the distribution of voxel intensity within the image region through commonly used and basic metrics, the histogram feature specifically describe the frequency distribution of voxel intensity. Histogram features on ADC maps have been shown to yield significant predictive performance in a multitude of studies—such as in Boca Petresc et al. [43], Lenoir et al. [27], Rodrigues et al. [44], and Fujima et al. [45]—and, as such, it is intuitive that they would reveal more about microstructural alterations in the primary lesion in this current study.

SmallDependenceLowGrayLevelEmphasis belongs to the Gray Level Dependence Matrix (GLDM), and it measures the joint distribution of small dependencies with lower gray level values. As such, it is an expression of the heterogeneity of the textural pattern, a parameter that has been shown, multiple times, to equate to higher malignancy [45,46,47].

The LASSO radiomics model applied to ADC maps, while showing a good AUC value, were slightly underperforming when compared to the post-contrast T1w model.

**Figure 6:**
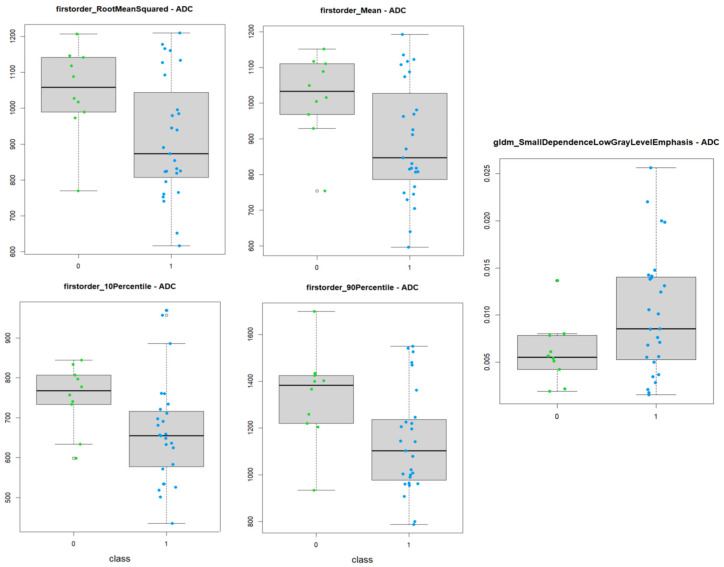
Box plot showing the different distribution of values of significant textural features among the two groups of patients on ADC maps.

The box plot chart for ADC maps shows the differences in the distribution of values among the two groups (Figure 6).

A LASSO model combining features from both post contrast T1w imaging and ADC maps was also elaborated, but it yielded significantly lower predictive power than the LASSO that was elaborated on the single dataset from one technique.

### 4.3. Limits and Future Perspectives

It is important to highlight some of the possible shortcomings of this study and how the authors managed to overcome them or alternatively point out the means to do so in future projects.

The low number of patients overall—and specifically of positives after RT-CHT treatment—is derived from the fact that the study dataset was collected as homogeneously as possible to reduce possible biases resulting from differences among patients enrolled. Secondly, as we know, HPV- and EBV-positive oropharyngeal and nasopharyngeal cancers tend to show a high response rate to medical therapy [24], and, therefore, it is difficult to study the recurrence of disease due to the—fortunately—relatively small number of patients. To this end, a decision was made to jointly study nasopharyngeal and oropharyngeal tumors, despite the different etiopathogenesis. The aim of this study was not the assessment of response to therapy dependent on the underlying pathogen, but rather how certain textural feature values found in tumoral tissue may indicate a more or less aggressive neoplasm and allow for better stratification of patients selected for exclusive RT-CHT treatment. Future studies with the two differentiated tumor classes are certainly necessary to strengthen and further validate the preliminary results of this study.

The strength of this study was, in the authors’ opinion, the use of a single MRI machine for the acquisition of all the data, in order to minimize heterogeneity that would otherwise be introduced from incongruent acquisition parameters, from the inherent variability between different vendors, or between different models from the same vendor. As this may currently limit the reproducibility of results on a different MRI machine, a comparison will therefore be useful to assess the effective application of the radiomic model on other machines.

## 5. Conclusions

Texture analysis on post-gadolinium T1 images and ADC maps could be a useful tool in the risk stratification of patients with OPC and NPC treated with RT-CHT. On post-contrast imaging, higher values in specific features associated with higher textural heterogeneity were correlated with worse outcomes, such as the recurrence/persistence of disease at follow-up. A similar result was obtained on ADC maps, with higher values in histogram features, associated with microstructural alteration and heterogeneity, correlating with an overall worse outcome. LASSO radiomics predictive models allowed for the distinction between patients with a recurrence/persistence of disease and patients that responded to therapy with strong predictive power.

## Data Availability

The data presented in this study are available on request from the corresponding author.

## Appendix A

**Table A1:** MRI acquisition parameters. Unenhanced scans included T1 and T2 sampling perfection with application-optimized contrasts using different flip angle evolution (SPACE) sequences with axial, coronal, and sagittal multiplanar reconstructions; axial T2 turbo spin echo; axial fat-saturated, echo-planar DWI spectral attenuated inversion recovery (SPAIR) with two b-values (b 50–800 s/mm^2^) and ADC maps. Enhanced scans carried out after intravenous gadolinium contrast agent (gadobutrol, 1 mL/10 kg, flow 3 mL/s followed by 20 mL saline flush) consisted of an axial T1 turbo spin echo and axial T1 volumetric interpolated breath-hold examination (VIBE) Dixon.

Sequence	Contrast Agent	Repetition Time (ms)	Echo Time (ms)	Slice Thickness (mm)	Interslice Gap (mm)	Field of View (mm)	Matrix	Acceleration Factor	Number of Signal Averaged	Band Width (Hz/Px)	Acquisition Time (min:sec)	Voxel Size
SPACE T1-w Sagittal	pre	500	7.2	0.9	-	229 × 229	230 × 256	2	1.4	630	5:47	0.9 × 0.9 × 0.9
SPACE T2-w Sagittal Fat-Sat	pre	3000	380	0.9	-	229 × 229	230 × 256	2	1.4	698	5:56	0.9 × 0.9 × 0.9
TSE T2-w Axial	pre	5050	117	3	0.9	210 × 190	261 × 484	2	3	191	2:23	0.5 × 0.5 × 3.0
SPAIR EPI-DWI Axial (b 50/800 s/mm^2^)	pre	4100	55	3	0.9	240 × 240	102 × 128	3	1	1608	3:09	1.6 × 1.6 × 3.0
VIBE T1-w DCE-PWI Axial; FA 5°, 15°	pre	4.65	1.66	3.5	0.7	250 × 226	139 × 132	3	1	390	1:04	1.3 × 1.3 × 3.5
TSE T1-w Axial	post	440	17	3	0.9	200 × 181	384 × 384	3	3	200	2:31	0.5 × 0.5 × 3.0
VIBE Dixon Axial	post	10	2.4	0.9	0.18	225 × 225	212 × 256	-	1	340	4:37	0.9 × 0.9 × 0.9
VIBE T1-w DCE-PWI Axial; FA 30°	post	4.65	1.66	3.5	0.7	250 × 226	139 × 132	3	1	300	4:17	1.3 × 1.3 × 3.5
